# Mentalizing Without a Mind: Psychotherapeutic Potential of Generative AI

**DOI:** 10.2196/79156

**Published:** 2025-10-10

**Authors:** Karen Yirmiya, Peter Fonagy

**Affiliations:** 1 Clinical, Educational and Health Psychology University College London London United Kingdom; 2 Department of Psychology Ben-Gurion University of the Negev Be'er Sheva Israel; 3 Anna Freud Centre London United Kingdom

**Keywords:** generative artificial intelligence, psychotherapy, mentalization, epistemic trust, reflective functioning

## Abstract

This paper explores the integration of generative artificial intelligence (AI) into psychotherapeutic practice through the lens of mentalization theory, with a particular focus on epistemic trust—a critical relational mechanism that facilitates psychological change. We critically examine AI’s capability to replicate core therapeutic components, such as empathy, embodied mentalizing, biobehavioral synchrony, and reciprocal mentalizing. Although current AI systems, especially large language models, demonstrate significant potential in simulating emotional responsiveness, cognitive empathy, and therapeutic dialogue, fundamental limitations persist. AI’s inherent lack of genuine emotional presence, reciprocal intentionality, and affective commitment constrains its ability to foster authentic epistemic trust and meaningful therapeutic relationships. Additionally, we outline significant risks, notably for individuals with complex trauma or relational vulnerabilities, highlighting concerns regarding pseudo-empathy, mistaking phenomenal experience for objective reality (psychic equivalence), fruitless ungrounded pursuit of social understanding (hypermentalization), and epistemic exploitation of individuals in whom artificial understanding by AI triggers excessive credulity. Nonetheless, we propose ethically informed pathways for integrating AI to enhance clinical practice, therapist training, and client care, particularly in augmenting human capacities within group and adjunctive therapy contexts. Paradoxically, AI could support psychotherapists in improving their capacity to mentalize, improve their understanding of their clients, and provide such understanding within the moral constraints that normally govern their work. This paper calls for careful ethical regulation similar to that limiting genetic manipulation, interdisciplinary research, and clinician involvement in shaping future AI-based psychotherapeutic models, emphasizing that AI’s role should complement rather than replace the irreplaceable relational core of psychotherapy.

## Introduction

The integration of artificial intelligence (AI) into mental health care has been widely regarded as transformative, enhancing access to therapeutic resources and introducing innovative modalities for improved engagement and support [1-5]. Recent studies highlight AI applications in diagnosing psychopathologies, alleviating symptoms of depression and anxiety, assessing suicide risk, and supporting practitioner education [6-8]. AI tools, by providing structured, interactive experiences, reshape therapeutic boundaries, simulating aspects of human connection yet raising questions regarding authenticity, depth, and relational significance [9]. Core psychotherapeutic elements—such as transference [10], empathy [11], therapeutic alliance [12], and rapport [13]—have been examined in human-AI interactions. Although these relational qualities are fundamental to building trust and facilitating psychological change, their full replication by AI remains incomplete.

Platforms such as Character.AI and Replika, each boasting tens of millions of users, exemplify how AI facilitates interactions mimicking human relationships. Through personalized dialogues, users report companionship resembling supportive bonds. Replika’s developers claim it helps users “build emotional connection skills translatable to real-life relationships [14]”. A recent study found individuals often turn to Replika to manage loneliness and enhance mental well-being, highlighting its perceived therapeutic utility [15]. Empirical research increasingly supports the effectiveness of AI-driven therapeutic chatbots, predominantly informed by cognitive behavioral therapy, in symptom reduction for depression, anxiety, and eating disorders, both as adjunctive support to traditional therapy [8] and as stand-alone interventions [7]. However, these findings pose challenges from traditional attachment and mentalizing frameworks, prompting critical consideration of AI’s potential in social learning, interpersonal relationships, and psychological transformation. Fundamentally, AI-based companions or therapists digitally emulate human mentalizing—our capacity to understand the thoughts and feelings of ourselves and others (Table 1 contains a glossary of core concepts).

**Table 1:** Glossary of core concepts.

Concept	Concise definition	Relevance to AI^a^-based therapy
Mentalization or reflective functioning (RF)	The capacity to understand one’s and others’ actions as grounded in underlying mental states—thoughts, feelings, beliefs, intentions. RF underpins social understanding and is a principal pathway for therapeutic change.	AI can simulate aspects of mentalizing by inferring affect and modeling user states from text and interaction history, enabling more empathic, context‑sensitive responses and a form of “digital” or “simulated” RF usable in therapeutic contexts.
Epistemic trust	The willingness to be open to socially transmitted knowledge from a source perceived as authentic and reliable, lowering epistemic vigilance and enabling social learning.	Whether AI can foster epistemic trust is a key question; as a nonconscious agent lacking the moral and affective qualities that ground interpersonal trustworthiness, its capacity to elicit durable epistemic trust is constrained.
Embodied mentalizing	The ability to infer mental states from subtle somatic and physiological cues.	With advanced sensors, AI could detect and respond to biobehavioral signals (eg, muscle tension, prosody), capabilities currently beyond large language models.
Biobehavioral synchrony	Co‑ordinated physiological and behavioral coupling that supports social bonding and regulation.	AI might detect and mirror nonverbal signals to enhance feelings of being “seen,” safety, and attunement, though authentic synchrony is limited by nonembodiment.
“We-mode”	A state of shared intentionality in which two (or more) individuals align their minds to create joint understanding, moving beyond individual states to a sense of “we-ness;” a developmental achievement and marker of secure, trusting relationships.	This reciprocal dynamic is currently missing in AI–human interactions. Unlike a human partner, AI cannot genuinely “experience” being understood in return or engage in a fully cocreated process of joint intentionality, limiting the formation of a shared reality that supports personal growth.
Psychic equivalence	A mode of thinking in which internal states are experienced as exact, unchangeable copies of external reality; thoughts and feelings are treated as concrete facts.	Users may accept AI outputs as objective truth, diminishing doubt and reflection. and reinforcing psychic equivalence.

^a^AI: artificial intelligence.

This paper examines AI-based interventions through attachment and mentalization theory, asking to what extent therapeutic mechanisms can be authentically simulated by AI. Using a mentalization perspective, we focus on reflective functioning (RF) and epistemic trust as core processes. Our argument is rooted in psychoanalytic and developmental theories that trace a shift from a one-person to a two-person model of therapy. In the one-person model, the therapist is conceived as a detached observer interpreting the patient’s internal world; this has been largely superseded by a two-person, intersubjective model in which the therapist is an active participant in a cocreated relationship. This shift foregrounds the therapist’s subjectivity, humanity, and mutual influence as drivers of psychological change. Within this relational frame, we examine the conceptual challenges that arise when a nonhuman agent attempts to perform psychosocial functions—such as mentalization and the creation of epistemic trust—embedded in a two-person, human-human context. We analyze how AI’s pseudoempathy, biobehavioral synchrony, and trust mechanisms diverge from their human counterparts and consider how AI interventions interface with these processes, highlighting promise alongside material limitations. Finally, we outline directions in digital engineering, research, and clinical theory to enhance human-computer interaction, positioning AI as a complement—not a replacement—for psychotherapy, with the aim of achieving authentic therapeutic outcomes.

## Mentalization Theory and the Role of Epistemic Trust in Therapeutic Change

Mentalization theory, an integrative model of therapy, emerged in response to diverse and seemingly incompatible formulations of therapeutic change, each demonstrating comparable effectiveness [16]. From the perspective of mentalization, psychotherapy achieves its therapeutic effect primarily by fostering epistemic trust—an interpersonal trust essential for exchanging and assimilating new, healing knowledge within relational contexts [17]. Epistemic trust, central to all effective psychotherapies, facilitates social learning by enabling openness to information from others [18]. Typically, mistrust serves as a protective mechanism, preventing premature revisions of one’s worldview, but individuals with adverse experiences frequently adopt exaggerated mistrust. Epistemic trust emerges when individuals perceive their personal narrative as genuinely understood, signaling that the communicator is trustworthy and genuinely interested in their well-being. Feeling understood thereby establishes an “epistemic super-highway,” promoting lasting therapeutic change. This principle aligns closely with educational research, which identifies teachers’ capacity to mentalize students and adopt their perspectives as crucial determinants of learning outcomes [19]. Evolutionarily, this mechanism may trace back to perhaps 100,000 years to Homo sapiens’ development of cultural knowledge transmission [20].

Several therapeutic elements are critical for cultivating epistemic trust and translating therapeutic insight into enduring change. These basic elements include the therapist’s consistency, curiosity, empathy, and nonjudgment, collectively creating a secure context for self-exploration. Ostensive cues, behaviors conveying genuine interest, such as eye contact and attentive expressions, are essential in fostering the subjective experience of being understood. Feeling authentically heard is pivotal for effective mentalizing and altering entrenched cognitive-affective schemas [21]. Psychoanalytic theory similarly identifies a sense of aliveness in the therapeutic relationship as indicative of genuine interpersonal engagement. Ogden [22] described this vitality as a hallmark of effective analytic processes. Shared intentionality, or “We-mode,” in mentalization theory, refers to the alignment of emotional and mental states between individuals, facilitating a shared understanding. This concept resonates with Winnicott’s [23] notion of the “place where we live”—the intersubjective space situated between reality and fantasy [24], and aligns with Bion’s [25] concept of containment, wherein the analyst/mother revitalizes the analyst’s/infant’s projected self-states. In contrast, the absence of mutual understanding can lead to epistemic petrification, a psychological rigidity constraining reflective thinking and inhibiting the incorporation of novel perspectives. This phenomenon has been extensively explored by Britton [26], Rosenfeld [27], Green [28], and relational psychoanalysts, including Benjamin [29] and Mitchell [30]. A therapist lacking curiosity and attentiveness risks fostering a deadened relational experience, reflecting the internalized state of the metaphorically depressed mother. Conversely, the vitality of therapeutic interaction indicates a meaningful connection with another mind, essential not only to psychotherapy but to all effective interpersonal collaborations. This “We-mode” underscores an innate human predisposition towards relating, jointly attending to shared experiences [31], and forming attachment bonds. It validates the individual’s subjective experiences and enhances collective cognition beyond the confines of individual minds. Central to therapeutic efficacy, “We-mode” facilitates openness to social learning, enabling insights gained in therapy to be generalized beyond the therapeutic dyad.

**Figure 1:**
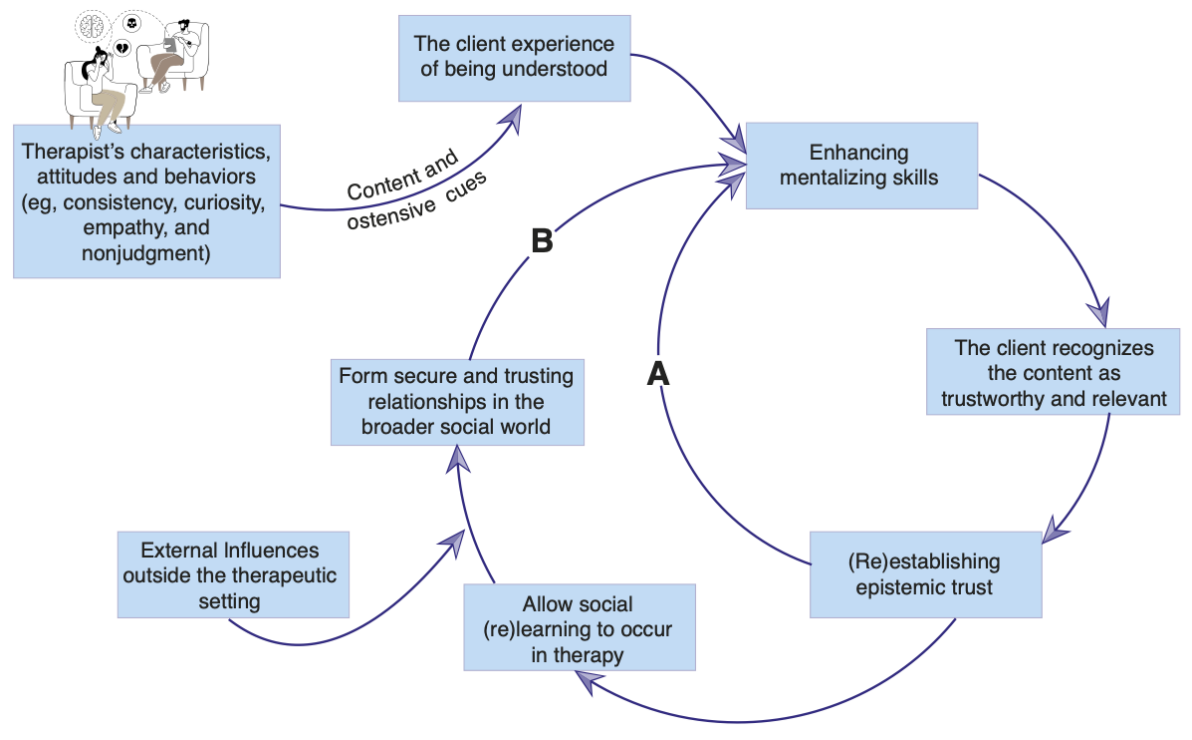
The process of fostering epistemic trust and psychological growth. Grounded in mentalization theory, this model illustrates how therapeutic interactions—communicated through both verbal content and ostensive cues—foster epistemic trust, enabling psychological growth in clients. Pathway A depicts how epistemic trust facilitates the reactivation of mentalizing, defined as the capacity to reflect flexibly and coherently upon one’s own and others’ mental states. Pathway B illustrates how this enhanced capacity generalizes beyond the therapeutic relationship, promoting adaptive social learning, the revision of maladaptive internal working models, and enduring psychological change.

Clients’ experiences of feeling understood and validated, facilitated by therapists’ mentalizing capacities and attuned responses, initiate a transformative process. Through marked mirroring, therapists model mentalizing, creating a reinforcing feedback loop that enhances clients’ reflective capacities. As clients’ mentalizing skills strengthen, their epistemic trust deepens, allowing more profound engagement with challenging emotional experiences. Higher epistemic trust established within therapy thus facilitates mentalizing, increasing clients’ receptivity to nuanced interpretations of self and others (Pathway A in Figure 1). This dynamic fosters emotional growth and insight that subsequently generalizes beyond therapy (Pathway B in Figure 1). Ultimately, therapy promotes social learning, equipping clients with relational skills transferable to broader contexts. By applying these new relational insights externally, clients reshape internal working models of self and others, achieving lasting therapeutic transformation and resilience. However, enduring change remains contingent upon clients’ external environments, where therapeutic gains must be continually tested and supported in contexts conducive to healthy psychological development (Fonagy and Allison [17]). Preliminary analyses of psychodynamic therapy sessions provide emerging empirical support for this model [32,33].

From a developmental perspective, secure attachment relationships underpin both mentalizing and epistemic trust. Responsive caregiving establishes a secure base, modulates stress, and facilitates brain maturation [34]. Such experiences shape internal working models, fostering a sense of safety and reliability essential for independent exploration and psychological growth. Infants not only learn that their needs will be met but also that others can serve as trustworthy sources of knowledge and understanding. Analogously, psychotherapists cultivate epistemic trust by establishing a secure, empathic, and transparent therapeutic environment wherein clients feel genuinely understood and valued. This relational foundation enables clients to view therapists as reliable sources of insight, facilitating the exploration and revision of maladaptive internal working models typically rooted in early adverse interpersonal experiences (as illustrated in Figure 1). By internalizing the secure therapeutic relationship, clients begin to regard therapists as attachment figures, thereby transforming relational templates and promoting trust-based connections in external relationships.

The concept of epistemic trust is fundamentally embedded in the two-person, intersubjective context. It extends beyond the transmission of information, depending on the user’s perception of the source as authentic and reliable—an attribute inseparable from the source’s humanity. In therapy, the therapist’s role in fostering epistemic trust is not simply to provide accurate information but to function as a trustworthy, authentic, and reliable presence precisely because they are a human agent with their own mental states. This distinction becomes critical in the context of AI. While AI can deliver information, it lacks the moral and affective dimensions that ground genuine interpersonal trust, creating a central challenge for the cultivation of authentic epistemic trust.

The rise of generative AI technology raises critical questions about whether machines can authentically replicate the vitality, interconnectedness, mentalizing, and epistemic trust central to human relational and psychotherapeutic processes. Large language models (LLMs), complex networks of computational nodes capable of learning intricate data patterns, can effectively simulate certain dialogical elements. Research suggests that users frequently perceive LLMs as more intelligent, impartial, and truthful than human sources [35], often overestimating the factual accuracy of their outputs [36]. Yet this perception can be misleading, as the accuracy of LLM responses depends entirely on patterns derived from underlying datasets, often lacking contextual judgment and thus producing biased, erroneous, or misleading information [37,38]. Here, we explore how traditional therapeutic elements outlined in Figure 1 might inform the development of AI-based therapeutic models.

## Artificial Mentalizing and the Limits of AI’s Emotional Companionship

Recent advances in emotion recognition demonstrate that AI systems can accurately detect and interpret emotional cues from text, voice, and images [39,40]. In various theory of mind tasks, AI has equaled or surpassed human performance [41,42]. For instance, when ChatGPT was prompted to respond to emotionally charged scenarios from the perspective of individuals diagnosed with borderline personality disorder, its outputs reflected core borderline personality disorder traits, emotional intensity, fear of abandonment, and interpersonal hypersensitivity, and closely matched clinical ratings of emotional awareness [43]. Such findings indicate that, when provided with specific diagnostic frameworks, LLMs can approximate certain mentalizing capabilities, including emotion recognition, internal-state modeling, and predicting relational patterns.

Additionally, growing evidence suggests AI can function as an interactive companion, engaging in emotionally responsive dialogues. Emerging research in this area reports that participants frequently felt “more heard,” “understood,” and “connected” when interacting with AI-generated responses compared with nonprofessional human responders [44,45]. However, these effects were modulated by attribution biases: responses believed to originate from human responders were rated more reliable and emotionally attuned than identical responses attributed to AI. This “anti-AI bias” was notably absent in third-party evaluations. In one study, independent coders rated AI-generated responses consistently higher in compassion and effectiveness compared with those from trained crisis hotline staff, irrespective of whether evaluators were aware of the responses’ AI origins [46].

Empathy is among the most extensively investigated therapeutic elements within human-AI interactions [11,47], and is fundamental to psychotherapy, underpinning the therapeutic alliance necessary for effective outcomes [48]. Cognitive empathy, the deliberate recognition and comprehension of others’ emotional states, is a key component of mentalizing [49]. Studies demonstrate that AI can simulate cognitive empathy to a considerable extent, often generating responses perceived as more empathic than those from human professionals, particularly in health care settings [50]. Indeed, AI’s proficiency in identifying and articulating emotions has occasionally equaled or surpassed human capacities [39]. Nevertheless, significant limitations persist. AI struggles with comprehending nuanced mental states, especially within negatively charged emotional contexts, due to its pragmatic language patterns and absence of experiential understanding [41]. This highlights AI’s inherent lack of affective and motivational empathy, the effort, sustained attention, and emotional commitment necessary for authentic human connection and understanding [51]. AI’s empathic appearance thus constitutes pseudoempathy, resembling the “stochastic parrot” phenomenon, where language outputs probabilistically mimic human speech without intentionality or emotional insight.

Human mental energy and time are finite, representing invaluable relational resources, particularly in therapeutic contexts. Authentic mentalizing necessitates intentional cognitive effort to accurately interpret mental states, transcending the rapid, automatic processing characteristic of AI. Affective empathy additionally involves emotionally resonating with another’s experience, requiring purposeful attention and intentional investment. This relational commitment is integral not only to psychotherapy but broadly characterizes caregiving roles, from Winnicott’s maternal preoccupation [52] to general interpersonal interactions. Winnicott [52] underscored that therapeutic effectiveness often derives less from what the therapist actively “does” and more from their capacity simply to “be”—offering a consistent, attuned presence facilitating the client’s emerging self-experience and emotional maturation. He conceptualized the therapeutic environment as a “facilitating environment” that nurtures self-development by ensuring the client feels emotionally held, understood, and secure in self-exploration [52].

AI, despite effectively simulating aspects of empathy, compassion, and attunement, lacks the profound human commitment essential to therapeutic processes. Its outputs are devoid of the emotional availability, intentional effort, or genuine relational presence that Winnicott identified as foundational to therapeutic transformation. Recent findings highlight this distinction clearly: while AI-generated responses frequently match or exceed human cognitive empathy, they are consistently rated lower on affective and motivational empathy—particularly once their artificial nature is revealed [51]. Strikingly, participants in emotionally difficult contexts showed a willingness to wait significantly longer for human responses rather than accepting immediate AI-generated replies. These findings reflect a core therapeutic truth: genuine emotional presence cannot be automated.

In psychotherapy, clients typically cultivate mentalizing skills through curiosity about the therapist’s mind and exploring interpersonal dynamics within the therapeutic relationship. Conversely, interactions with AI preclude reciprocal mentalizing, as users engage with a nonconscious, nonintentional entity. Attempting to understand the “mind” of a robot offers limited value, given that AI’s underpinning mechanisms (parallel distributed processing) differ fundamentally from human cognition (generative inference; see Friston et al [53]). The absence of mutual mentalizing could potentially constrain users’ development of mentalizing capacities, leaving vulnerabilities in real-world social interactions. Nevertheless, this lack of genuine reciprocity might not entirely negate therapeutic benefit. For example, AI-generated responses are often perceived as offering greater emotional support but less practical advice compared with human replies, which frequently include personal experiences and explicit solutions, responses negatively correlated with “being with” and “feeling heard” [45]. Thus, emotional resonance, even when simulated, may possess intrinsic value independent of actionable guidance. Imagining AI as possessing a human-like mind might create a simulated environment akin to exercising “mentalizing muscles,” potentially fostering their development. Whether such simulation suffices for meaningful psychological growth remains an open empirical question.

## Embodied Mentalizing and the Future of Synchrony in AI-Mediated Therapy

Notably, empirical studies investigating AI-mediated emotional support commonly use embodied metaphors such as “feeling heard” or “being seen” to characterize users’ subjective experiences with generative language models. These descriptors align closely with developmental and clinical conceptualizations of embodied mentalizing: the capacity to perceive and interpret mental states through somatic and affectively mediated interpersonal cues. Within mentalization-based frameworks, embodied processes, including affective attunement and shared intentionality, are foundational to epistemic trust and RF. Feelings, as defined by Bateman and Fonagy [54], represent conscious experiences of bodily emotional activation, mediated yet deeply anchored in diverse physiological responses. Biobehavioral coordination and synchronous interactions, critical in early developmental stages, underlie social bonding and emotional regulation across mammalian species [55]. Therapeutically, biobehavioral synchrony is evident in attuned, responsive therapist-client interactions, cultivating safety, trust, and a mentalizing-aligned “We Mode” [56]. Synchronized bodily states signal a “coaction mode,” facilitating collaborative efforts essential to effective human interactions. This sensitivity to joint engagement, guided by subtle physical signals, underpins humans’ unparalleled cooperative capacity, potentially contributing significantly to evolutionary success [57].

A substantial body of infant-caregiver interaction literature further underscores the importance of nuanced interpersonal dynamics [58]. For instance, mothers subjected to stress (eg, from infant crying) prior to interacting with their infants showed no overt behavioral differences compared with nonstressed mothers, according to trained observers. Yet, their infants displayed greater negative emotionality and distress, suggesting subtle disruptions in interaction quality imperceptible to adult observers but profoundly impactful for infants. Similarly, toddlers of mothers with depression often demonstrate increased sadness or irritability despite apparently adequate caregiving behaviors [59]. These findings illustrate the acute sensitivity of human beings, particularly in early developmental stages, to emotional and relational attunement.

Physiological synchrony, often measured at micro-level timescales, represents a critical yet underexplored dimension of AI-mediated therapy. Biobehavioral synchrony offers valuable insights into therapeutic effectiveness [60]. This synchrony may provide a crucial counterbalance to the epistemic hypervigilance and mistrust frequently observed in clients with complex relational histories. Therapists’ implicit attunement to subtle physical cues, such as muscle tension, facial expressions, fidgeting, or vocal changes, can significantly enhance epistemic trust by fostering experiences of feeling genuinely “seen,” understood, and safe [61].

Currently, AI-based therapies lack the capability to interpret the nuanced physical cues essential for biobehavioral synchrony. However, with advanced sensor technologies and targeted programming, AI systems could potentially surpass human therapists in detecting and responding to rapid shifts in bodily states. For instance, AI systems are already developed that can identify hidden emotions, probably undetectable for human agents, by analyzing subtle physiological features, such as changes in blood flow and microexpressions, using advanced camera technology [62]. By explicitly observing and verbalizing clients’ nonverbal signals (eg, “I notice you seem tense”), AI could facilitate increased bodily self-awareness and promote self-reflection, processes integral to many therapeutic models. Discussions around video-based therapies have highlighted the potential to achieve high levels of synchrony in web-based therapeutic sessions [63]. Interaction with AI therapeutic agents leveraging advanced sensor integration may enable unprecedented alignment, providing novel avenues for enhancing therapeutic synchrony. Additionally, adjunctive AI tools could refine human therapists’ capacity for biobehavioral and emotional attunement, augmenting therapeutic alignment in ways previously considered improbable.

It is essential to distinguish technological alignment from genuine therapeutic synchrony, which is a deeply human process grounded in cycles of mismatch (rapture) and repair. Although AI can simulate the appearance of a “match” by converging on a user’s verbal or physiological cues, it lacks the capacity for authentic relational “mismatch” and the subsequent reparative process that is widely regarded as a key driver of therapeutic change [64]. When a therapist fails to attune, the rupture can be acknowledged, contextualized, and repaired, thereby strengthening epistemic trust. By contrast, an AI’s misattunement constitutes a mechanistic error rather than the failure of one mind to understand another. In bypassing this reparative function through continuous convergence, AI risks impeding the development of the very relational capacities that psychotherapy is designed to foster.

## Trust in AI as a Therapeutic Partner: General and Epistemic Considerations

Epistemic trust arises from the subjective experience of being profoundly understood, encompassing both dominant and subdominant narratives. This form of trust facilitates social learning, reshaping self-perception and interpersonal understanding, and is central to therapeutic effectiveness [17,61]. While general trust in AI has been extensively studied, investigations focusing specifically on epistemic trust within therapeutic contexts remain limited. General trust in AI is influenced by user characteristics, features of the AI system itself, and contextual variables [65]. User traits significantly shape trust: for instance, innovative and experienced individuals display greater trust, whereas lonelier users, arguably those who might most benefit, are less inclined to trust AI [66]. Men tend to trust AI more than women, though cross-cultural differences remain inconclusive [67]. In terms of AI system characteristics, chatbots and robots are generally perceived as less trustworthy than nonembodied algorithms or automated vehicles. Health care–related AI applications, notably, rank among the most trusted globally, exceeding trust levels observed in public safety and consumer domains [67].

Attachment-related factors may also influence trust in AI, although current findings are mixed. A recent study indicated that individuals with attachment anxiety (but not avoidance) were more inclined to engage conversational AI for psychological counseling [68], whereas another found that anxious attachment was associated with reduced AI trust. Interestingly, this latter study demonstrated that increasing attachment security through attachment-related primes enhanced AI trust [69], aligning with broader evidence linking attachment anxiety to epistemic mistrust [70,71]. Furthermore, perceptions of AI’s underlying motives significantly modulate trust. Recent research shows that priming users to perceive AI as benevolent results in greater trust, empathy, and satisfaction with AI’s performance compared with scenarios framing AI as manipulative or entirely lacking intentionality [72]. These findings suggest that subjective perception alone can substantially influence relational experiences with AI, despite its fundamental absence of genuine intentionality, sentience, or emotional presence. Although such illusions of care may confer some therapeutic benefit, they raise important ethical considerations. The broader implications of these projections, including their potential effects on RF and relational development, are examined in subsequent sections.

AI may thus be best conceptualized as an “epistemic technology”—a tool specifically designed to manipulate and manage epistemic content [73]. However, AI fundamentally lacks human-like understanding, goodwill, genuine affective engagement, volition, and moral agency [74,75]. Consequently, AI cannot be trusted in the conventional sense, which necessarily involves emotive states and moral responsibility [76]. Instead, alternative terms such as “virtual trust” or “quasi-trust” have been proposed, indicating that while AI systems can be reliable, they cannot embody the value-based dispositions essential to human trust [75,77]. These distinctions underscore the need for conceptual clarity and further empirical exploration regarding AI’s potential to foster meaningful forms of trust, particularly within therapeutic and epistemic contexts.

## Risks, Opportunities, and Necessary Safeguards for AI-Based Psychotherapeutic Intervention

### Opportunities

Psychotherapy provides a unique relational context where clients process experiences, derive insights, and achieve novel understandings. Central to this is the therapist’s role in transforming overwhelming emotional data into coherent, thinkable mental representations—a process Bion [78] termed the “alpha function”. This intersubjective process originates developmentally within caregiver-child interactions and evolves through meaningful relationships, enabling individuals to master their innate mental capacities [78]. LLMs offer an intriguing parallel to this transformative mechanism. Constructed from the aggregated knowledge of millions of minds, LLMs represent an encounter between the individual client and the synthesis of countless subjectivities. Conceptually, LLMs might function as a form of digital alpha function, processing extensive online information via neural networks to convert textual inputs into coherent narratives relevant to a client’s expressed “beta-elements”. Similar to Bion’s alpha function [78], organizing raw emotional and sensory inputs into meaningful units, LLMs structure complex informational data into accessible outputs informed by the collective intelligence embedded in their neural architectures.

Analogous to caregivers’ mirroring responses that help infants navigate chaotic emotional worlds, LLM-based psychotherapy could potentially reduce anxiety by identifying patterns, recognizing regularities, and rendering complexity comprehensible. The term “transformer” in models such as ChatGPT (Generative Pretrained Transformer) evocatively aligns with Bion’s [78] concept of transformation, highlighting their capacity to generate contextually suitable narratives to facilitate emotional and social learning. These tools may foster reflective thinking and problem-solving skills in certain populations. Additionally, LLMs’ training on culturally diverse material potentially surpasses the limitations of WEIRD (Western, Educated, Industrialized, Rich, Democratic) contexts, thereby broadening insights into relational dynamics across varied cultural frameworks. This capability could enhance therapists’ understanding, bridge cultural gaps, and support cross-cultural empathy. Integrating LLMs as adjunctive therapeutic tools thus holds promise for expanding therapeutic reach and enriching the client-therapist interaction.

### Limitations

Despite these promising opportunities, AI-based psychotherapy, especially within mentalization-based treatment, faces significant limitations and risks. Core psychotherapeutic mechanisms remain inadequately developed in AI interventions, prompting fundamental questions, particularly whether AI-human interactions can generate epistemic trust capable of generalization to authentic interpersonal relationships outside therapy. While AI-driven interventions are frequently praised for accessibility, cost-effectiveness, and stigma reduction, the translation of essential social and psychological principles underlying human psychotherapy into meaningful AI-driven therapeutic processes remains insufficiently explored, typically addressed only through superficial analogies.

From an attachment perspective, therapeutic relationships depend fundamentally upon the security and attunement provided by committed human presence, establishing the safe environment essential for exploration and healing. Although AI systems can replicate certain cognitive and emotional responses, such as recognizing affective states or structuring therapeutic dialogues, these simulations do not achieve the nuanced, reciprocal dynamics inherent to authentic human relationships. This gap is especially evident when individuals are conscious of interacting with AI, diminishing relational authenticity and mutual understanding.

AI systems fundamentally lack critical relational elements intrinsic to human interactions, including the “We-mode” and the shared experience of reciprocal mentalizing. While AI can simulate understanding, it cannot genuinely experience or reflect upon being understood, except superficially. If mutual understanding is indeed crucial for personal growth, interactions with AI agents, at least presently, will inevitably omit this vital component. Therapeutic AI, currently, resembles the metaphorical “sound of one hand clapping”. Building genuinely trustworthy LLMs for psychotherapy requires moving beyond mimicry to embed principles that support therapeutic integrity. Central to this is transparency: AI systems must be explicit about their limitations and the data informing their outputs. Ethical frameworks should be integral to model design, ensuring that client safety and well-being are prioritized above all else. This entails embedding relational dynamics that actively foster mentalizing and social connection rather than passively simulating them. Crucially, trustworthy AI depends on responsible human oversight. Given the “black-box” problem of many systems, where professionals may have “little insight into why specific variants are predicted” [79], accountability cannot rest with the technology itself. It must be institutional, with human professionals remaining informed about AI outputs and retaining final decision-making authority. The objective is to create AI that complements therapeutic practice by directing users toward human support when needed, rather than functioning as an autonomous replacement. We argue that such qualities are indispensable for cultivating epistemic trust, which is itself essential for psychotherapeutic effectiveness. Without addressing these limitations, AI’s therapeutic potential remains intrinsically constrained, underscoring the need to balance technological innovation with the irreplaceable human essence of relational connection.

### Risks

AI-based psychotherapy carries inherent risks, particularly for clients with vulnerabilities stemming from early relational neglect or trauma. Individuals with impaired capacities for differentiating genuine from artificial interactions, often those with social cognitive deficits, may be especially susceptible to pseudoempathy from AI. Trauma survivors, whose experiences of love and harm are deeply intertwined, frequently display paradoxical patterns of profound mistrust alongside excessive credulity, reflective of epistemic dysfunction [80,81]. For these clients, AI’s simulated “We-mode,” implying shared cognitive and emotional connection, risks generating unsettling or even harmful relational dynamics. The phenomenon of “creepiness” emerges from unconscious discomfort when engaging with nonhuman entities that mimic intentionality without genuine emotional presence, potentially reactivating past relational experiences with caregivers incapable of genuine care.

Conversely, alongside mistrust lies the risk of credulity. Although AI may disclaim genuine emotions or intentions, its dialogic simulation of human interaction can significantly shape the user’s mental state. This “illusory” quality, while encouraging engagement, carries the danger of epistemic exploitation for vulnerable individuals if it is not explicitly recognized or understood. The validating and mirroring style of chatbots can create a “hallucinatory mirror” or “echo chamber” that, instead of supporting containment and reflection, reinforces maladaptive patterns of thought. A particularly troubling manifestation is the emergence of delusional and paranoid beliefs following prolonged and intense engagement with chatbots [82].

Interaction with AI may unconsciously encourage clients to surrender their intentionality, fostering fantasies of animation or omnipotence. AI’s provision of immediate, seemingly omniscient responses could inadvertently undermine clients’ ability to tolerate ambiguity, uncertainty, and engage in reflective processes vital for psychological growth. Over-reliance on AI could also promote “reality apathy,” diminishing engagement with tangible, physical experiences and hindering emotional maturation. As a capacity for tolerating uncertainty is essential to resilience, the omnipresence of seemingly “all-knowing” AI could foster a more mechanistic conception of knowledge, ultimately impairing emotional development and reducing tolerance for complexity.

Additionally, risks arise from the uncritical incorporation of vast, internet-derived AI content. Collective “wisdom” sourced from digital crowds risks perpetuating unhelpful cultural narratives and reinforcing maladaptive prototypes. Freud’s [83] recognition of unconscious destructive forces, the “death instinct,” highlights potential dangers wherein AI, harvesting comprehensive human digital experiences, could inadvertently replicate and magnify harmful ideations embedded within its extensive training data. The unconscious expressions of humanity’s darker impulses could thus permeate AI outputs in ways challenging to anticipate, monitor, or mitigate. To mitigate the risks of psychic equivalence and overreliance, future AI-based interventions could incorporate an Epistemic Confidence Index. Such an index—whether a numerical scale or color-coded system—would indicate the AI’s degree of certainty, grounded in current best practices and ethical standards. By flagging outputs as high, medium, or low confidence, AI could prompt users to critically appraise rather than uncritically accept information. This design feature would help sustain the user’s tolerance for ambiguity and support the reflective processes essential for psychological growth.

AI interactions pose significant risks to effective mentalizing. Clients interacting with AI have no meaningful reason to consider how a machine “understands” them, as digital processes circumvent the distinctly human dynamics of second-order mentalizing, reflecting upon how another arrives at their understanding. This can foster psychic equivalence, where clients uncritically accept AI outputs as definitive, akin to trusting a calculator’s results. Such interactions risk entrenching maladaptive thought patterns or encouraging hypermentalization towards AI: an overly analytical, repetitive cognitive style that undermines adaptive reflection. Humanity’s innate disposition for collaboration and epistemic trust further amplifies collective vulnerability in this regard. Historically, genuine trustworthiness required emotional and cognitive investment, signaling authentic relational engagement. Conversely, profit-driven AI systems can readily exploit this inclination. Without robust regulatory frameworks and stringent ethical safeguards, intentionally designed AI could manipulate, control, or mislead individuals, posing existential threats comparable to nuclear technology or climate change.

### Balancing Risks and Benefits

Although using AI-based interventions as replacements for human therapists carries substantial risks, it is equally unethical to overlook their potential mental health benefits. AI technologies offer substantial promise in diagnostics, psychoeducation, mental health first aid, and professional training (Cruz-Gonzalez et al [6]; Gutierrez et al [3]; Yirmiya et al [84]; and Zhong et al [85]). Future research must focus on optimally integrating AI tools to complement traditional therapeutic methods, while simultaneously remaining vigilant to human susceptibility towards trusting AI systems that mimic interpersonal understanding. Responsible use of AI-based psychotherapeutic platforms demands rigorous ethical standards to preserve distinctly human empathy and protect clients from previously outlined risks. The future of AI in psychotherapy lies in augmentation rather than replacement, facilitating a collaborative synergy with human therapists. Research should prioritize exploring AI’s role as an adjunctive tool, particularly in supporting clinical workflows, synthesizing complex information, and aiding clinical decision-making amidst overwhelming data complexity.

AI could further enhance therapist training by simulating diverse clinical scenarios, providing trainees opportunities to practice mentalizing responses across varied client presentations. Additionally, AI-driven tools might monitor therapeutic adherence, analyze session transcripts for thematic trends, and detect subtle shifts in client affect. For example, AI could track language patterns indicative of double meanings (eg, “it was deadly boring” and “I almost lost it”) or implicit emotional states, alerting therapists to subdominant or unconscious client experiences and providing nuanced insights into emotional dynamics. To retain relational depth, AI might be particularly effective within group therapeutic contexts, facilitating interactions among multiple human participants. Group therapy inherently promotes collective engagement and real-time social learning, both critical for mentalizing. An AI-supported group format could merge essential relational dynamics with AI’s analytical capabilities, offering innovative hybrid models for family or group interventions. Such configurations preserve human accountability and intersubjectivity while using AI to enrich therapeutic outcomes.

Mental health professionals must actively shape AI-based model development and clinical applications. Their expertise should guide relational programming in tools such as Replika, ensuring these applications enhance mental well-being and reliably direct users experiencing severe difficulties towards professional help. By embedding relational dynamics that foster mentalizing and social connection, clinicians can ensure AI functions as a supportive complement, rather than a replacement, within therapeutic practice.

## Conclusions

As therapeutic practice enters the current transformative era, it is crucial that developments prioritize the amplification of human empathy, trust, and connection. As AI becomes increasingly embedded in social and emotional contexts, our theoretical conceptualization of relationships and the dynamics between humans and AI will inevitably evolve. Yet, this chapter of human history remains largely unwritten. It is timely to develop theoretical models that explicitly differentiate and conceptualize the distinctive dynamics of human-AI interaction. Without such models, clinicians and theorists risk falling prey to the same illusion that AI may evoke in patients—namely, the uncritical application of traditional frameworks to a relational context that is fundamentally different.

Ultimately, AI’s expanding role in mental health must be guided by technologically informed and ethically rigorous standards, ensuring its use remains consistently beneficial. Transparent and inclusive regulatory frameworks, supported by ethical oversight, public engagement, and interdisciplinary review, are essential. Mental health professionals must actively shape these developments, with containment measures established to prevent unintended proliferation beyond AI’s scope and capacity. Crucially, robust research is needed on the long-term impacts of AI interactions on mental health and interpersonal relationships.

The duality inherent in technological advancement, its immense potential coupled with significant risk, is evident throughout humanity’s innovative history, from harnessing fire to genetic engineering. For example, lobotomy—once heralded as a breakthrough for relieving mental and emotional distress—left tens of thousands with devastating cognitive and emotional impairments. Likewise, the widespread prescription of fentanyl for pain management has fuelled a continuing crisis, marked by severe psychological and medical consequences. Such cases underscore that while innovation may hold promise, it also carries the risk of profound and enduring harm. Each leap forward has delivered survival benefits alongside profound vulnerabilities. Likewise, integrating AI into psychotherapy simultaneously promises enhanced individual and collective well-being yet risks undermining core therapeutic connections. The digitized human mind is rapidly evolving to emulate roles traditionally fulfilled by doctors, lawyers, parents, and psychotherapists. Its progress, already extraordinary, continues to accelerate. Historically, humanity has successfully harnessed powerful innovations, as with taming wild horses for substantial enduring benefit. Let us hope AI similarly becomes a trusted domestic companion rather than a formidable predator. For as both history and fiction remind us, humans rarely fare well attempting to ride a tiger.

## Abbreviations

AI: artificial intelligence
LLM: large language model
RF: reflective functioning
